# Bacteriotherapy for the treatment of intestinal dysbiosis caused by *Clostridium difficile* infection^☆^

**DOI:** 10.1016/j.mib.2013.06.009

**Published:** 2013-10

**Authors:** Blessing O Adamu, Trevor D Lawley

**Affiliations:** Bacterial Pathogenesis Laboratory, Wellcome Trust Sanger Institute, Hinxton, Cambridge CB10 1SA, UK

## Abstract

Faecal microbiota transplantation (FMT) has been used for more than five decades to treat a variety of intestinal diseases associated with pathological imbalances within the resident microbiota, termed dysbiosis. FMT has been particularly effective for treating patients with recurrent *Clostridium difficile* infection who are left with few clinical options other than continued antibiotic therapy. Our increasing knowledge of the structure and function of the human intestinal microbiota and *C. difficile* pathogenesis has led to the understanding that FMT promotes intestinal ecological restoration and highlights the microbiota as a viable therapeutic target. However, the use of undefined faecal samples creates a barrier for widespread clinical use because of safety and aesthetic issues. An emerging concept of bacteriotherapy, the therapeutic use of a defined mixture of harmless, health-associated bacteria, holds promise for the treatment of patients with severe *C. difficile* infection, and possibly represents a paradigm shift for the treatment of diseases linked to intestinal dysbiosis.

## Highlights

• Antibiotics damage the intestinal microbiota and disrupt colonization resistance predisposing us to recurrent *C. difficile* infection (CDI).
• Faecal microbiota transplantation (FMT) is a promising treatment for recurrent *C. difficile* infection.
• Mixtures of beneficial bacteria known as bacteriotherapy should be developed for treatment of CDI and other diseases linked to dysbiosis in the intestinal microbiota.

**Current Opinion in Microbiology** 2013, **16**:596–601

This review comes from a themed issue on **Antimicrobials**

Edited by **Robert EW Hancock** and **Hans-Georg Sahl**

For a complete overview see the Issue and the Editorial

Available online 15th July 2013

1369-5274/$ – see front matter, © 2013 The Authors. Published by Elsevier Ltd. All rights reserved.

**http://dx.doi.org/10.1016/j.mib.2013.06.009**

## Introduction

The human intestine is inhabited by a diverse and abundant community of microorganisms, collectively termed the intestinal microbiota, that plays crucial roles in our development and sustenance [1]. Proper functioning and homeostasis of our intestine relies on an intimate and symbiotic relationship between our mucosal surface, the microbiota and its metabolic by-products. Homeostasis is characterized by a diverse microbiota that produces a variety of metabolites such as short-chain fatty acids (SCFAs), and this is coupled to a lack of pathology associated with specific T-cell subsets [2].

Pathological imbalances in the intestinal microbiota, referred to as dysbiosis, are increasingly linked to intestinal diseases. Disturbances in the intestinal microbiota caused by infections and antibiotics have profound effect on the microbiota's composition and function and can predispose the individual to antibiotic-associated diarrhoea (AAD). In the last decade, the incidence of morbidity and mortality from *Clostridium difficile* infection (CDI), the leading cause of AAD, increased largely due to the emergence and global spread of fluoroquinolone-resistant variant of *C. difficile* (characterized as genotype BI/NAPI/027) [3]. *C. difficile* is continuously evolving in response to antibiotic selection and other ribotypes (such as 017 and 078) have also been identified in humans and animal sources that could also spread globally [4,5]. First line treatment for severe *C. difficile* infections include metronidazole or vancomycin although in 15–35% of these cases, a recurrence (relapse or reinfection) follows the cessation of antibiotic therapy.

Faecal microbiota transplantation (FMT) has been mainly used as an alternative treatment for patients with persistent, recurrent *C. difficile* infection and involves the restoration of the intestinal microbiota through instillation or engraftment of homogenized faecal suspension from a healthy donor [6^•^]. FMT has also been utilized in the treatment of diseases associated with intestinal dysbiosis such as inflammatory bowel diseases (IBD; manifested as Crohn's disease and ulcerative colitis), irritable bowel syndrome (IBS) and obesity [6^•^]. This review focuses on the use of FMT for the treatment of recurrent CDI, the potential for designing a mixture of ‘harmless’ bacteria for the treatment of CDI infection and applications to other diseases. We refer to this clinical application as ‘Bacteriotherapy’.

## The human intestinal microbiota in health and disease

The human intestinal microbiota contains >1000 bacterial species, as well as other understudied organisms like Archaea, eukaryotic organisms and viruses, that collectively encode 150 times more unique genes than the human genome [7^••^,8,9]. Intestinal bacteria consist mainly of strict anaerobes (>99%) and are involved in a variety of functions beneficial to the host, including immunological development and stimulation, SCFA production from the breakdown of dietary fibre, conversion of xenobiotics to less harmful substances, maintaining intestinal epithelial integrity and generation of nutrients and vitamins [10,11].

**Figure 1:**
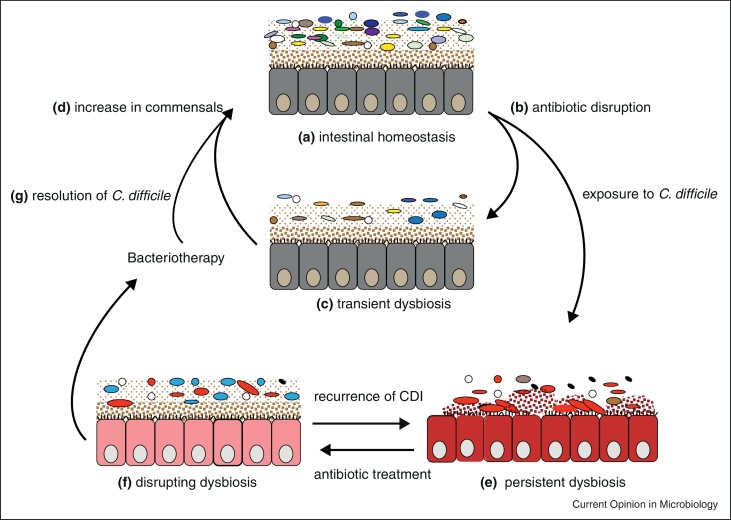
A proposed model for recurrent *C. difficile* infection and the restoration of the intestinal microbiota through FMT or bacteriotherapy. Intestinal homeostasis **(a)** is characterized by large microbial diversity in the microbiota and health-associated metabolites. Antibiotic exposure disrupts the microbiota **(b–c)** by destroying the microbial community leading to reduction in the diversity and loss of colonization resistance. The microbiota usually expands in diversity **(d)** after antibiotics are stopped to restore diversity. Antibiotic disruption makes individuals hyper-susceptible to *C. difficile* colonization potentially leading to chronic infection and persistent dysbiosis **(e)**. After treatment of CDI with antibiotics such as vancomycin, further microbiota disruption **(f)** occurs potentially leading to recurrent CDI after discontinued use of the antibiotic. FMT or bacteriotherapy disrupts intestinal dysbiosis leading to resolution of CDI **(g)** and increase in species diversity (d) and restores intestinal homeostasis.

An important function of our microbiota is colonization resistance, through acting as a barrier against pathogen colonization or overgrowth of resident opportunistic bacteria present at low levels [2,10,12]. These processes are made possible due to the presence of abundant and diverse microorganisms competing with an invading bacterium directly for niches and nutrients or through production of antibacterial products like bacteriocins [2] (Figure 1). Other factors such as diet, hygiene, lifestyle, host genetics and immune status influence the bacterial groups present in the intestinal microbiota to promote intestinal homeostasis and colonization resistance [13].

Various diseases have been associated with dysbiosis in the intestinal microbiota such as AAD, IBD, IBS, asthma and obesity [14–17]. In addition, certain pathogens, such as *Salmonella* Typhimurium and *Citrobacter rodentium*, exploit intestinal inflammation to subvert colonization resistance in mice [18,19]. Inflammation of the intestinal mucosa caused by enteric pathogens leads to dysbiosis and a decrease in the species diversity of the intestinal microbiota, allowing opportunistic pathogens to flourish at the expense of the commensal or beneficial microbes [7^••^].

## Effects of antibiotics on the intestinal microbiota

Numerous studies in humans and animals have investigated the effects of various antibiotic classes on the intestinal microbiota [14,20]. Generally, antibiotics deplete the overall organismal abundance and drastically alter the composition leading to a number of metabolic shifts such as decreased production of SCFAs due to reduced carbohydrate fermentation [14,21,22]. After cessation of antibiotic treatment, the microbiota typically recovers in abundance and diversity but may not return to the original community structure as some species may be missing [20] (Figure 1).

The extent of perturbation and damage depends on the particular antibiotic used and the degree of resistance within the community [14]. For example, vancomycin is active against both commensal and pathogenic Gram-positive bacteria leading to intestinal dysbiosis [23–26]. Antibiotic usage can also lead to an increase in antibiotic resistant organisms such as vancomycin-resistant *enterococci*, methicillin-resistant *Staphylococcus aureus* and transfer of antibiotic resistance genes among the microbial community [23,25,27]. The long-term consequence of antibiotic exposure on host health is poorly understood. However, during the short-term (days to weeks), the host may become hyper-susceptible to certain infections and antibiotic-associated diarrhoea.

## *Clostridium difficile* diarrhoea

Antibiotic perturbation of the microbiota predisposes the host to pathogen colonization and overgrowth and potentially diarrhoeal disease caused by bacteria such as *Klebsiella oxytoca*, *Clostridium perfringens*, pathogenic *Escherichia coli* and *C. difficile* [28]. *C. difficile*, an anaerobic, spore-forming Gram-positive bacterium, is the leading cause of AAD in hospitalized patients [3,29]. The incidence of CDI in the last decade has increased leading to a public health burden with estimated economic costs of at least $1.5 billion per year in the USA alone [30].

Major risk factors for CDI include prolonged antimicrobial use, exposure to a healthcare environment and advanced age [29]. *C. difficile* produces 2 toxins encoded by *tcdA* and *tcdB* (though some variants also produce a third binary toxin) that are known to facilitate pathogenesis [31]. *C. difficile* colonization is associated with different outcomes ranging from asymptomatic carriage, mild/chronic diarrhoea, fulminant colitis to toxic megacolon and even death [29,32^•^] (Figure 1).

*C. difficile* produces highly resistant and transmissible spores that can potentially persist in the gut of infected individuals or contaminate skin or environmental surfaces. These spores are resistant to antibiotics and can re-colonize and germinate after antibiotic therapy leading to recurrent disease [29,33]. Asymptomatic carriers are also a source of spores that can promote transmission and persistence at both the hospital and global levels [5].

Standard treatments for CDI involve the use of antibiotics such as metronidazole and vancomycin [34,35]. However, 15–35% of these patients usually experience recurrent disease, leaving few treatment options [36] (Figure 1). Many patients become dependent on vancomycin (oral, tapered and/or pulsed) to maintain remission and this has implications for the intestinal microbiota. Chang *et al.* used 16S rRNA gene clone libraries to analyse the faecal microbiota of 7 patients with initial and recurrent CDI [21]. This study demonstrated that patients with recurrent CDI had decreased proportional abundance of Bacteroidetes and increased Proteobacteria and Verrucomicrobia [21]. However, a new antibiotic, fidaxomicin was found to have lower rates of recurrence of *C. difficile* infection associated with the non-epidemic strain compared to vancomycin, an effect that is attributed to its lower activity against commensal and beneficial gut microbes [26].

## FMT for treatment of recurrent CDI

Because of the recent increase in the rates of recurrence and severity from epidemic *C. difficile* strains, there has been renewed interest in the use of FMT for the treatment of recurrent CDI. In this process, homogenized faeces from a healthy donor is infused through colonoscopy, enema or nasogastrically to an individual with *C. difficile* disease to restore the intestinal microbiota and thereby eradicate CDI [37–39] (Figure 1). The donor, usually a healthy individual/relative, is screened for contagious pathogenic agents such as *Salmonella* spp., *Staphylococcus aureus*, *C. difficile* and HIV and other infections or inflammatory conditions as previously described [32^•^].

The intestinal microbiota of *C. difficile* patients treated with FMT is characterized by expansions in species diversity characterized by an increase in *Bacteroides*, *Roseburia* and *Faecalibacterium* and a reduction in *Enterobacteriaceae* [40] (Figure 1). Recently, Hamilton and co-workers analysed the faecal microbiota of 3 patients treated by FMT using frozen donor sample from the same individual and an increase in Bacteroidetes and Firmicutes was observed [41]. The Bacteroidetes were represented by greater abundance of the families *Bacteroidaceae*, *Rikenellaceae* and *Porphyromonadaceae* and members of the Firmicutes were represented by *Lachnospiraceae*, *Ruminococcaceae* and unclassified Firmicutes [41]. These families are typically found in abundant numbers in the intestines of healthy individuals, suggesting that key groups of health-promoting bacteria are associated with the displacement of CDI and restoration of homeostasis in treated patients.

Several studies that employed FMT for the treatment of recurrent CDI have reported success rates of 86–100% [32^•^,40–42]. van Nood and colleagues recently published the first clinical trial to directly compare FMT with vancomycin only or vancomycin with bowel lavage for the treatment of recurrent CDI [43^••^]. Remarkably, FMT resolved 13 of the 16 cases (81%) after first infusion compared to only 31% for vancomycin and 23% for vancomycin with bowel lavage [43^••^]. On second infusion, 2 of the 3 failed cases had resolution of CDI leading to an overall success rate of >90% in this trial [43^••^]. The success rates observed in these studies highlight the importance of having a healthy and diverse intestinal microbiota and should establish FMT as a viable clinical option for diseases associated with intestinal dysbiosis.

Presently, there is no global standardized protocol for FMT as the process is largely dependent on the centre performing the FMT. However, it is pertinent to note that in 2011, the Faecal Microbiota Transplantation Workgroup proposed standard guidelines for treatment of CDI with FMT such as patient inclusion/exclusion criteria, donor selection and screening, preparation and administering of faecal samples [32^•^].

## Rational design of a defined bacteriotherapy

**Figure 2:**
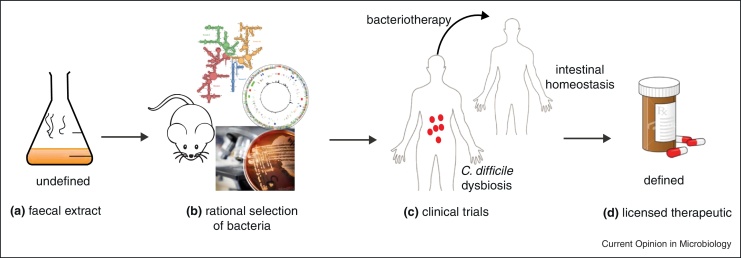
Generic model to create a standardized, defined bacteriotherapy mixture for treatment of patients with severe CDI. Culturing and genomic profiling of faecal samples from healthy donors and CDI patients **(a)** could potentially identify candidate bacteria that can be tested *in vivo* for safety and efficacy. Whole genome sequencing will define the bacteria and provide a basis to determine phylogenetic position within the microbiota community **(b)**. Clinical trials **(c)** will be required to test efficacy of bacteriotherapy mixture in diseased humans with severe CDI before widespread clinical use **(d)**.

The use of undefined faecal samples for FMT creates a barrier for widespread clinical use, mainly because of the amount of time needed to prepare and screen donor samples, patient safety issues, non-standardization of the treatment procedure and general doctor and patient aversion [6,44]. Therefore, there is an unmet clinical need to design a combination of harmless, health associated bacteria as a viable therapeutic option (Figure 2). Over two decades ago, Tvede and Rask-Madsen demonstrated that simple mixtures of 10 bacteria isolated from healthy faecal samples can resolve recurrent *C. difficile* as effectively as whole faecal transplants [45^••^]. More recently, a mixture of 33 bacterial species isolated from a healthy donor was used to eradicate CDI in two patients [46^••^] These studies have pioneered the concept of ‘Bacteriotherapy’ in humans but have yet to meet the scrutiny of regulatory agencies during the development a pharmaceutical product [47].

Experimental studies are also providing insight into the mechanisms of successful FMT. A recently developed murine infection model for *C. difficile* used a mixture of 6 defined bacteria to cure mice infected with the epidemic *C. difficile* 027 strain [48]. During the resolution of infection, at least 4 out of 6 bacterial strains colonized the mice post-transplant and many low level commensal bacteria present during disease expanded to increase the microbiota diversity during disease resolution [48]. Whole genome sequencing was used to establish the phylogeny of these therapeutic bacteria within the broader microbiota and also rule out the possibility that they code for known virulence factors. An important study, Reeves *et al.* have used novel culturing methods to identify a single *Lachnospiraceae* strain that can suppress *C. difficile* infection in mice [49]. Studies into the basic mechanisms of *C. difficile* suppression should guide the rational selection of candidate bacteria for bacteriotherapy development (Figure 2).

Furthermore, it will be important to identify the microbial differences between healthy individuals and patients with severe *C. difficile* disease, as this could guide the selection of the bacterial species from the healthy donors (Figure 2). It may be necessary to conduct a retrospective study to identify the shifts in the intestinal microbiota of patients’ post-FMT from several studies that have been published in order to identify what bacterial groups are present after FMT. Though, it is worth noting that personalized responses to FMT have been previously observed [50] and this could be due to factors such as diet, genetics and lifestyle that influence the microbiota structure and function.

## Emerging applications of bacteriotherapy

Bacteriotherapy has potential applications for other diseases associated with intestinal dysbiosis such as IBD and IBS. Borody and colleagues used FMT to treat 6 patients with refractory ulcerative colitis and follow up at 1–13 years post-FMT showed no clinical evidence of ulcerative colitis [51]. Similarly, Duplessis *et al.* utilized FMT to treat a patient with severe Crohn's disease complicated by refractory CDI [52]. Obesity is another condition that could potentially benefit from FMT [17,53]. FMT from lean to obese individuals led to increased insulin sensitivity in the patients compared to the control group [54].

## Conclusion

The diversity of the human intestinal microbiota is key to a number of biological processes that ensure the wellbeing of an individual. Alterations caused by long-term antibiotic use and infections are detrimental to the host as seen in CDI. FMT is increasingly being accepted as a treatment for recurrent CDI, but large, randomized double-blinded studies are needed. However, beyond FMT, bacteriotherapy using standardized mixtures of beneficial bacteria could potentially be used in the future for the treatment of recurrent CDI and other diseases associated with dysbiosis in the intestinal microbiota such as IBD, IBS and obesity.

## References and recommended reading

Papers of particular interest, published within the period of review, have been highlighted as:

• of special interest
•• of outstanding interest

## Figures and Tables

Figure modified from [48].
